# High density lipoprotein inhibited group II innate lymphoid cells proliferation and function in allergic rhinitis

**DOI:** 10.1186/s13223-022-00681-3

**Published:** 2022-05-11

**Authors:** Shengli Gao, Qingxiang Zeng, Yinhui Zeng, Yiquan Tang, Wenlong Liu

**Affiliations:** Department of Otolaryngology, Guangzhou Women and Children’s Medical Center, Guangzhou Medical University, No. 9, Jinsui Road, Guangzhou, 510623 China

**Keywords:** HDL, Allergic rhinitis, ILC2

## Abstract

**Background:**

More and more studies had suggested that dyslipidemia was closely related to allergic diseases. High density lipoprotein (HDL) often plays anti-inflammatory and anti-oxidative roles by suppressing immune cell chemotaxis and activation. We aimed to explore the role of HDL in the regulation of group II innate lymphoid cells (ILC2) in allergic rhinitis (AR).

**Methods:**

The blood lipid levels and their correlation with symptom scores of 20 AR subjects and 20 controls were analyzed. Purified ILC2 were stimulated by HDL and cytokines production were examined by enzyme-linked immunosorbent assay (ELISA) and flow cytometry. The mRNA levels of GATA binding protein 3(GATA3) and retinoid-related orphan receptor α (RORα) expressed by ILC2 were detected using reverse transcription polymerase chain reaction (RT-PCR).

**Results:**

HDL level was significantly lower in AR than controls and correlated with the symptom scores. The serum HDL levels were negatively related to the increased number of ILC2, IL-5^+^ ILC2, and IL-13^+^ ILC2 in AR patients. HDL decreased the number of ILC2 and type II cytokines levels significantly by inhibiting expression of GATA3 and RORα.

**Conclusions:**

Our data provide preliminary evidence that HDL may play a negative role in ILC2 inflammation in AR, suggesting that HDL may serve as promising treatment target in AR.

## Introduction

Allergic rhinitis (AR), featured with allergic inflammation of the upper airway, impairs the quality of life of more than 10% world population [1]. Traditionally, AR is considered as eosinophilic and augmented type 2 immune inflammation [2].

Accumulating evidence confirmed the correlation between dyslipidemia and allergic diseases, such as AR, [3–5] asthma, [4, 6] and atopic dermatitis (AD). Dyslipidemia regulates both innate and adaptive immune reaction by releasing pro-inflammatory cytokines, which contribute to the activation and polarization of T-helper 2 (Th-2) and Th17 as well as inhibiting interleukin-10 (IL-10) cytokine production [7].

HDL is composed of several apolipoproteins, such as apoA-I, apoA-II, apoCs, apoE, serum amyloid A (SAA). HDL often plays anti-inflammatory [8] and antioxidant roles by suppressing immune cell chemotaxis and activation [9, 10]. Trakaki’s study found that AR significantly altered the composition and function of HDL, suggesting a new correlation between HDL metabolism and allergy [11].

Group II innate lymphoid cells (ILC2) are a group of cells lacking markers of T cell, B cell, natural killer cell, et al. ILC2 can initiate allergic inflammation by producing Th2 cytokines after stimulation of IL-33, IL-25 or thymic stromal lymphopoietin (TSLP). Several studies had shown that the frequency of ILC2 elevated significantly in AR patients. However, the interaction between HDL and ILC2 was not understood.

In this study, we aimed to assess (1) the correlation between HDL and the proliferation and function of ILC2, (2) the regulation of HDL on ILC2 by cell culture.

## Methods

### Patients

A total of 40 subjects (> 18 years old) included 20 AR and 20 controls were enrolled in our study. The inclusion criteria for AR patients included: (1) confirmed diagnosis of AR as described by the Allergic Rhinitis and its Impact on Asthma (ARIA) guideline, (2) at least 1 year history, (3) typical symptoms such as sneezing, blocked nose, itchy nose and runny nose, (4) allergic to at least one inhalant allergen (dust mites, pets, molds, cockroach, etc.) confirmed by specific IgE measurement (Phadia ImmunoCAP, Sweden). The exclusion criteria included: (1) the presence of other allergic diseases, such as asthma, dermatitis, (2) acute or chronic rhinosinusitis, (3) pregnancy or breastfeeding, (4) previous treatment with immunotherapy, (5) with immunologic disease, tumors, or chronic infection, (6) use of systemic corticosteroids or anti-histamine drugs in the past 2 weeks, and (7) with diabetes, hypertension, myxedema, hypothyroidism, obesity, liver and kidney diseases and other diseases which may affect blood lipid levels. Normal subjects had no history of allergy and positive allergen test. The exclusion criteria for controls were similar to that of AR. Our study obtained approval and written informed consent from local ethics committee boards.

### Total nasal symptom scores

The nasal symptoms severity questionnaire was completed. The nasal symptoms including sneezing, runny nose, itchy nose, and blocked nose were rated between 0 and 3 scale (0, none; 1, mild; 2, moderate; and 3, severe).

### Blood lipid measurement

Venous fasting blood were obtained for assaying serum total cholesterol (TC), low-density lipoprotein (LDL), high-density lipoprotein, and triglyceride (TG) levels. The blood lipids were determined using a Cobas Integra 400 (Roche, Switzerland).

### Effects of HDL on ILCs

Peripheral blood mononuclear cells (PBMCs) were purified using density-gradient centrifugation from of AR and controls. PBMCs (1.0 × 10^6^ cells/mL) were incubated in RPMI 1640 supplemented with 10% heat-inactivated fetal calf serum and treated by human HDL-DiI (Biotrend, FL) for 24 h at 37 °C.

For determination of ILC2s, lineage markers-FITC, FceRI-APC, CD45-APC/Cy7, CRTH2-PE, CD127-PE-Cy7 antibody (BD Bioscience, NJ) were used for staining. Lin^−^ FceRI CD45^+^ CRTH2^+^CD127^+^ cells were defined as ILC2s. The proliferation of ILC2 was determined using tritiated thymidine incorporation.

### Real-time PCR

Total RNA was extracted from stimulated PBMCs by TRIzol (Invitrogen, US). RNA was reverse-transcribed for cDNA synthesis. PCR reaction was performed using real-time PCR detection system (BioRad). The relative levels of target genes was normalized to GAPDH housekeeping gene using 2-ΔΔCt method. The primers were listed as follows: GATA3 sense, 5ʹ-GCGGGCTCTATCACAAAATGA-3ʹ, antisense, 5ʹ-GCTCTCCTGGCTGCAGACAGC-3ʹ; RORα sense, 5-AAGGAGCCAGAAGGGATGAAC-3ʹ, antisense, 5ʹ-GGAACA ACAGACGCCAGTAAG-3ʹ; GAPDH sense, 5ʹ- AGCCACATCGCTCAGACAC-3ʹ, antisense, 5ʹ- GCCCAATACGACCAAATCC -3ʹ.

### Enzyme-linked immunosorbent assay (ELISA)

The concentration of IL-5, IL-13 were examined using ELISA kits (R&D systems, USA). The sensitivity of cytokines was: IL-5, 3.9 pg/mL, IL-13, 125 pg/mL.

### Statistical analysis

Prism (GraphPad Software 8.0) were used for analysis with *P* values lower than 0.05 as statistically significant in all analyses. The Kruskal–Wallis H test or nonparametric Mann–Whitney U test was done. Spearman rank was done for correlation analysis.

## Results

### Blood lipids levels

The demographic information of subjects is summarized in Table 1. The levels of LDL, TC, TG between control and AR group had no significant difference, whereas the HDL level in AR group was significantly lower compared with control group. Moreover, the HDL level was significantly correlated with the symptom scores of AR.

**Table 1:** Demographic characteristic of AR patients and normal controls

Groups	AR	Control
Number	20	20
Age (years)	24.6 ± 8.8	27.1 ± 9.2
BMI	21.3 ± 3.5	21.9 ± 3.8
Sex (Male:Female)	12:8	11:9
TC (mg/dl)	151.09 ± 36.78	128.45 ± 27.33
LDL (mg/dl)	67.17 ± 19.65	75.31 ± 18.11
HDL (mg/dl) TG (mg/dl)	31.18 ± 6.52* 78.24 ± 19.15	68.75 ± 16.29 83.26 ± 11.45

Compared with control group, *P* < 0.05

*AR* allergic rhinitis, *TC* total cholesterol, *LDL* low-density lipoprotein cholesterol, *HDL* high-density lipoprotein, *TG* triglyceride

### Correlation between HDL and ILC2

**Fig. 1:**
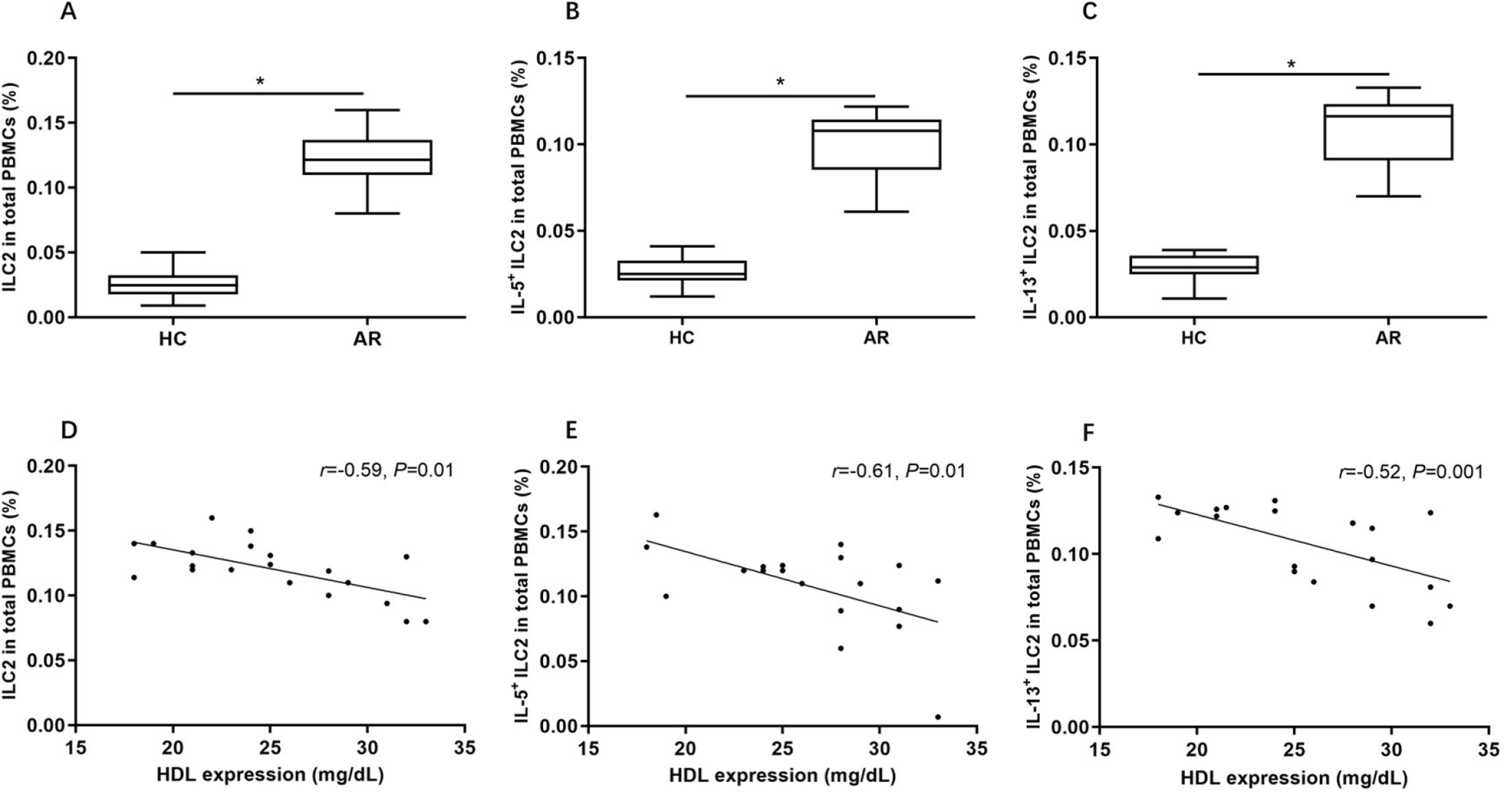
The proportion of ILC2, IL-5^+^ILC2, and IL-13^+^ILC2 cells between allergic rhinitis and controls as well as their correlation with HDL expression **A**–**C** The proportions of ILC2, IL-5^+^ILC2 and IL-13^+^ILC2 between allergic rhinitis and controls. **D**–**F** Negative correlation between HDL expression and the proportions of ILC2, IL-5+ILC2, IL-13+ILC2 in AR. HDL, high density lipoprotein. AR, allergic rhinitis. HC, healthy control. *P<0.05.

The number of ILC2, IL-5^+^ ILC2, and IL-13^+^ ILC2 cells were significantly higher in AR than controls (Fig. 1). The serum HDL concentration was negatively correlated with the number of ILC2, IL-5^+^ ILC2, IL-13^+^ ILC2 (Fig. 1). However, serum TC, TG and LDL were not correlated with the number of ILC2, IL-5^+^ ILC2, and IL-13^+^ ILC2 cells.

### ILC2s inhibited by HDL

**Fig. 2:**
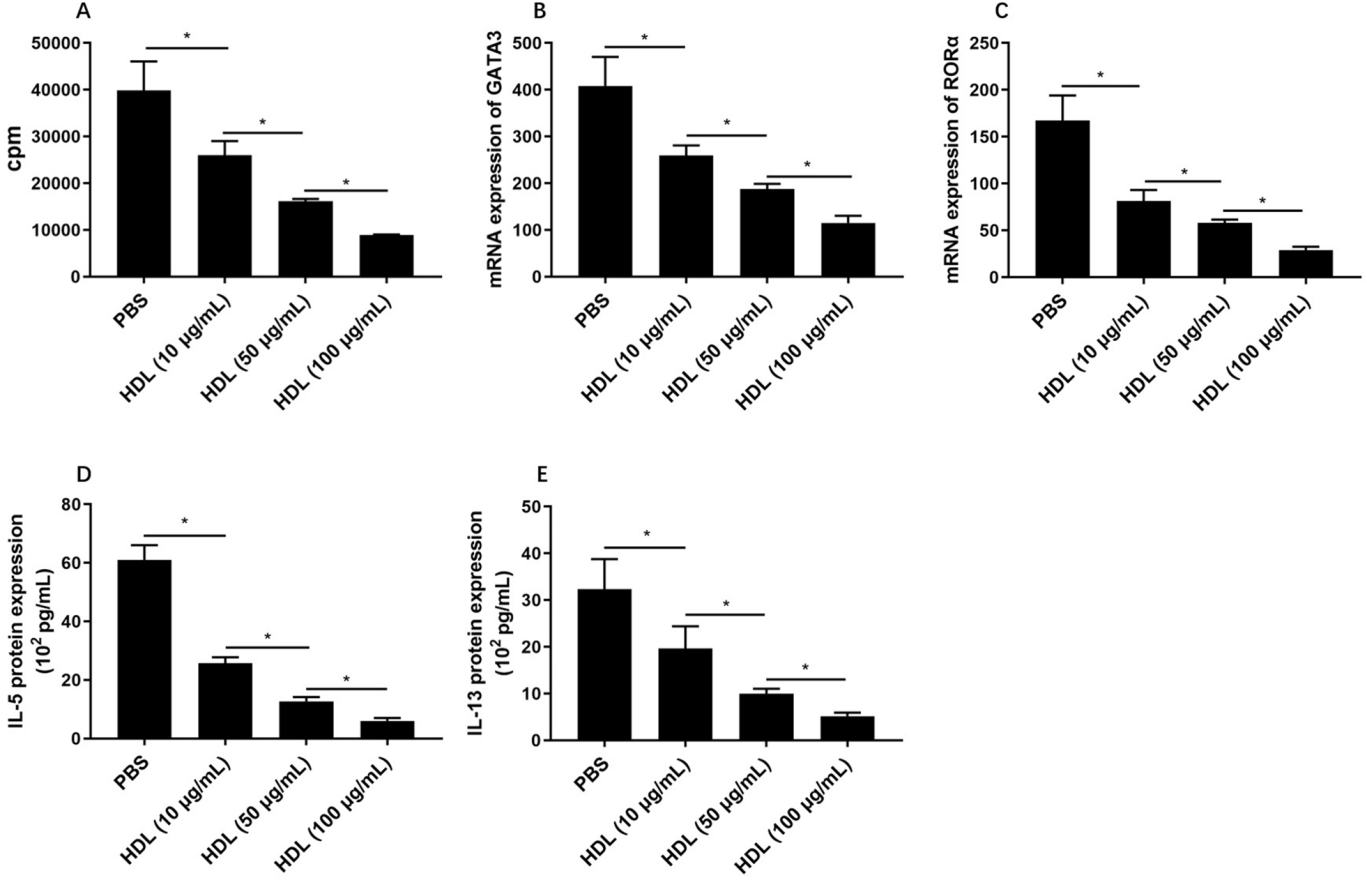
The ILC2 cell proliferation and cytokine expression regulated by HDL. **A** Proliferation of ILC2 was assessed by using tritiated thymidine incorporation under HDL stimulation. **B**, **C** The mRNA levels of GATA3 and RORα by ILC2 determined by PCR. **D**, **E** The IL-5 and IL-13 protein expression by ILC2 assessed by ELISA. Three independent tests were performed for every experiment. HDL, high density lipoprotein. *P < 0.05.

HDL decreased the number of ILC2 significantly when they were cocultured together accompanied by down-regulating mRNA level of GATA3 and RORα by ILC2 (Fig. 2). The protein levels of IL-5 and IL-13 were inhibited after HDL treatment (Fig. 2).

## Discussion

Over last years, the prevalence of obesity, allergic sensitization and atopic diseases increased significantly [12]. Accumulating evidence suggested that metabolism is correlated with chronic airway inflammation [13]. More and more studies have concentrated on the correlation of between allergic diseases and various lipids.5 For example, HDL-C has an anti-inflammatory effect by regulating T-cell activation [14, 15]. A meta-analysis including ten studies showed elevated LDL level and decreased HDL levels in asthma subjects compared with controls [6]. Vinding et al. 's[4] study found that LDL and TG levels were correlated with the occurrence of asthma, airway obstruction and aeroallergen sensitization. Recently, hypercholesterolemia has been associated with Th2-oriented inflammation in allergic diseases, such as asthma and related disorders [16–18]. However, the role of dyslipidemia in the regulation of ILC2 mediated inflammation was not reported in the previous studies.

Consistent with previous study, our study also confirmed that.

Down-regulated HDL level in AR children was correlated with disease severity [19, 20]. Moreover, HDL was related to the proliferation and function of ILC2, suggesting that HDL is engaged in the regulation of ILC2.

Next, we purified and cultured ILC2 to investigate the role of HDL. We found that HDL inhibited the proliferation and type II cytokine production by ILC2, which was regulated by GATA3 and RORα pathway. These data confirmed that HDL regulated ILC2 directly. Similarly, Li’s study demonstrated that coculture of ox-LDL and peripheral blood mononuclear cells (PBMC) contributed to increased number of ILC1s and decreased number of ILC2s in a dose-dependent manner [21]. Moreover, their studies showed that ILC1s and ILC2s were more susceptible to ox-LDL-mediated alterations in ACI patients compared with controls [21]. These results suggested that lipids may play different roles in different backgrounds.

Our study had following limitations. First, the sample size was limited. Therefore, large sample and multi-center study was needed in future. Secondly, we did not introduce mice model in this study to prove our in vitro results. Thirdly, the effect of HDL on ILC2 in obese AR patients was needed in future study. Fourth, ILC2 are believed to have a role in homeostasis of adipose tissue. Whether the response of ILC2 to HDL is just a reflection of this property and how HDL exert its effect on ILC2 at different site of body needs animal studies to explore, especially HDL gene related Knockout mice.

In summary, our study provided preliminary evidence for the first time that HDL inhibited the proliferation and function of ILC2 from peripheral blood in AR by in vitro studies, further in vivo studies were needed to explore the effect of HDL on ILC2 as well as the causal relationship between allergic inflammation and metabolic disorders.

## Data Availability

The datasets used and/or analysed during the current study are available from the corresponding author on reasonable request.

## Abbreviations

HDL: High density lipoprotein
ILC2: Group II innate lymphoid cells
AR: Allergic rhinitis
ELISA: Enzyme-linked immunosorbent assay
GATA3: GATA binding protein 3
RORα: Retinoid-related orphan receptor α
RT-PCR: Reverse transcription polymerase chain reaction
AD: Atopic dermatitis
Th-2: T-helper 2
IL-10: Interleukin-10
SAA: Serum amyloid A
TSLP: Thymic stromal lymphopoietin
TC: Total cholesterol
LDL: Low-density lipoprotein
HDL: High-density lipoprotein
TG: Triglyceride
PBMCs: Peripheral blood mononuclear cells

## References

[1] Katelaris CH, Lee BW, Potter PC (2012). Prevalence and diversity of allergic rhinitis in regions of the world beyond Europe and North America. Clin Exp Allergy.

[2] Han X, Krempski JW, Nadeau K (2020). Advances and novel developments in mechanisms of allergic inflammation. Allergy.

[3] Ouyang F, Kumar R, Pongracic J (2009). Adiposity, serum lipid levels, and allergic sensitization in Chinese men and women. J Allergy Clin Immunol.

[4] Vinding RK, Stokholm J, Chawes BL, Bisgaard H (2016). Blood lipid levels associate with childhood asthma, airway obstruction, bronchial hyperresponsiveness, and aeroallergen sensitization. J Allergy Clin Immunol.

[5] Schäfer T, Ruhdorfer S, Weigl L (2003). Intake of unsaturated fatty acids and HDL cholesterol levels are associated with manifestations acids and HDL cholesterol levels are associated with manifestations of atopy in adults. Clin Exp Allergy.

[6] Peng J, Huang Y (2017). Meta-analysis of the association between asthma and serum levels of high-density lipoprotein cholesterol and low-density lipoprotein cholesterol. Ann Allergy Asthma Immunol.

[7] Yang WQ (2016). Study of the relationship between IL-10 polymorphism and serum lipoprotein levels in Han Chinese individuals. Genet Mol Res.

[8] Barter PJ, Nicholls S, Rye KA (2004). Antiinflammatory properties of HDL. Circ Res.

[9] Catapano AL, Pirillo A, Bonacina F (2014). HDL in innate and adaptive immunity. Cardiovasc Res.

[10] Trieb M, Wolf P, Knuplez E (2019). Abnormal composition and function of high-density lipoproteins in atopic dermatitis patients. Allergy.

[11] Trakaki A, Sturm GJ, Pregartner G (2019). Allergic rhinitis is associated with complex alterations in high-density lipoprotein composition and function. Biochim Biophys Acta Mol Cell Biol Lipids.

[12] Baumann S, Lorentz A (2013). Obesity—a promoter of allergy?. Int Arch Allergy Immunol.

[13] Dixon AE, Holguin F (2019). Diet and metabolism in the evolution of asthma and obesity. Clin Chest Med.

[14] Welty FK, Alfaddagh A, Elajami TK (2016). Targeting inflammation in meta-bolic syndrome. Transl Res.

[15] Manti S, Leonardi S, Panasiti I (2017). Serum IL-10, IL-17 and IL-23 levels as “bioumoral bridges” between dyslipidemia and atopy. Cytokine.

[16] Baldán A, Gomes AV, Ping P (2008). Loss of ABCG1 results in chronic pulmonary inflammation. J Immunol.

[17] Yeh YF, Huang SL (2004). Enhancing effect of dietary cholesterol and inhibitory effect of pravastatin on allergic pulmonary inflammation. J Biomed Sci.

[18] Sevelsted A, Stokholm J, Bønnelykke K (2015). Cesarean section and chronic immune disorders. Pediatrics.

[19] Yon DK, Lee SW, Ha EK (2018). Serum lipid levels are associated with allergic rhinitis, nasal symptoms, peripheral olfactory function, and nasal airway patency in children. Allergy.

[20] Sheha D, El-Korashi L, AbdAllah AM (2021). Lipid Profile and IL-17A in allergic rhinitis: correlation with disease severity and quality of life. J Asthma Allergy.

[21] Li Q, Liu M, Fu R (2018). Alteration of circulating innate lymphoid cells in patients with atherosclerotic cerebral infarction. Am J Transl Res.

